# Simple Route to Obtain Nanostructured CeO_2_ Microspheres and CO Gas Sensing Performance

**DOI:** 10.1186/s11671-017-1951-x

**Published:** 2017-03-06

**Authors:** Edgar R. López-Mena, Carlos R. Michel, Alma H. Martínez-Preciado, Alex Elías-Zuñiga

**Affiliations:** 1 Escuela de Ingenieria y Ciencias, Tecnologico de Monterrey, Campus Monterrey, Ave. Eugenio Garza Sada 2501, 64849 Monterrey, N.L. México; 2 Departamento de Física, CUCEI, Universidad de Guadalajara, 44410 Guadalajara, Jal. México; 3 Departamento de Ingeniería Química, CUCEI, Universidad de Guadalajara, 44410 Guadalajara, Jal. México

**Keywords:** CeO_2_, Coprecipitation, Microspheres, Gas sensor

## Abstract

In this work, nanostructured CeO_2_ microspheres with high surface area and mesoporosity were prepared by the coprecipitation method, in absence of a template. The reaction between cerium nitrate and concentrated formic acid produced cerium formate, at room temperature. Further, calcination at 300 °C yielded single-phase CeO_2_ microspheres, with a diameter in the range 0.5–2.6 μm, the surface of these microspheres is completely nanostructured (diameter about 30–90 nm). CeO_2_ microspheres were used to fabricate a sensor device, and it was tested for intermediate CO gas concentrations (200–800 ppm). The detection of 200 ppm carbon monoxide was observed at 275 °C, with a response time of 9 s, using an applied frequency of 100 kHz. The detection of changes on the CO gas concentration was studied at different temperatures and applied frequencies. The results revealed a reproducible and stable gas sensing response.

## Background

Cerium oxide (CeO_2_) is one of the most reactive rare earth oxides, with unique physical and chemical properties, such as oxygen storage capacity, oxygen deficiency, and electronic conductivity [1–4]. These characteristics have allowed CeO_2_ to be widely used in catalysis, energy storage, biosensors, among others [5–10]. In recent years, CeO_2_ has also been investigated as gas sensor; Izu et al. used the mist pyrolysis method to prepare CeO_2_ at 700 °C. Here, a powder with an average particle size of 200 nm and a random network structure was obtained [11]. In this study for a comparative purpose, CeO_2_ with average particle sizes of 200 and 2000 nm was used to fabricate a resistive oxygen gas sensor. They concluded that the response time (8 s) for the sensor with smaller particle size (200 nm) was approximately one-tenth of the time taken by the sensor with a larger particle size (2000 nm) at 712 °C. Moreover, when the sensor kinetics is controlled by the diffusion of oxygen vacancies, the response time varies with the particle size. These conclusions were supported in a previous work reported by Beie et al. [12]. Xie et al. who obtained CeO_2_ nanoparticles (10–15 nm) at 500 °C using a solvothermal method [13]. They used these nanoparticles to prepare a humidity sensor device. In a humidity range of 11–98% (relative humidity, RH), the response and recovery times were 10 and 3 s, respectively. In addition, two sensing mechanisms were reported. At low RH, the process is given by conduction and polarization of the grains of CeO_2_, whereas at high RH, the conduction process is attributed to decomposition and polarization of the absorbed water.

In recent decades, there has been an ever-increasing demand for gas sensors. CO has attracted the attention for its monitoring due to the health risks caused by prolonged exposure to this gas. Some of the characteristics of CO are colorless, odorless, tasteless, and non-irritating but lethal. CO is a poisonous gas produced by the incomplete burning of carbon-based fuels. At concentrations below 200 ppm, this gas induces slight headache and dizziness. In a range between 200 and 400 ppm, the exposure to CO promotes a slight headache and loss of judgment. At 800 ppm, the health risk is greater (dizziness, nausea, and convulsions), being lethal at 12800 ppm with only 3 min of exposure [14].

Several semiconductor materials have been tested as CO gas sensor; SnO_2_ is one of the most tested materials for this purpose [15–18]. CeO_2_ has also been tested as a CO sensor though low (<100 ppm) or high (>1000 ppm) is usually reported. At low CO gas concentrations, Itoh et al. used zirconium-doped cerium oxide to prepare a sensor device [19]. This device was tested in CO concentration in a range between 0.1 and 2 ppm though no details for response time were given. Durrani et al. used CeO_2_ commercial pellets to obtain thin films at 500 °C as sensor device [20]. To study the CO gas sensing response, operation temperatures in the range 300–500 °C and gas concentrations between 500 and 50,000 ppm were tested. With these conditions, they found that the optimum operating temperature was around 390 °C and the response and recovery time were 45 and 25 s, respectively. Izu et al. studied CeO_2_ thick films using Pt/alumina as a catalyzer; different synthesis temperatures were tested in the range 800–1100 °C [21]. The best result was obtained at 450 °C using 5000 ppm. The response time was 2 s with an average particle size of 58 nm.

The most important parameters involved in the detection of gas species are the operating temperature, sensitivity, selectivity, and long-term stability. These can be improved in a material by decreasing its particle size to the nanometer scale, as well as an increase in the porosity [22]. According to what several authors have reported [23–25], the sensitivity of a material is reciprocal to the grain size as long as the depletion region extends over the grain. Thus, there is an importance to get a grain size twice smaller than the depletion layer thickness, and this occur when the grain size is in a nanometer scale (*D* < 2 *L*, *L* = depletion layer). As the grain size is smaller, a higher surface area is obtained. To maximize the specific surface area, the particles should be loosely packed; then a very small contact area between each other is obtained. This is obtained with spherical or semispherical morphology. However, to improve the gas sensing performance, an excellent connectivity between the grains is necessary. In this point, the synthesis temperature plays a key role. If the material is synthesized at high temperature, the grain size increases as well as the connectivity between them. However, the surface area decreases, and given that the gas sensing mechanism is surface area dependent, we will have an effect in this phenomenon. For porous materials, the gas is easy to be diffused into the bulk, which promotes the rapid response and recovery of the sensor. In addition, when a material is mesoporous (2 nm < pore radii < 50 nm), the gas transport occurs by molecular diffusion, whereas if the material is microporous (pore radii < 1 nm), the gas transport becomes a surface diffusion. So, it is also important to control the morphology.

With this purpose, CeO_2_ has been synthesized by numerous methods where different chemical agents were used. To mention some, Xu et al. synthesized broom-like porous CeO_2_ hierarchical architecture by hydrothermal method [26]. For this synthesis, dihydrate trisodium citrate, urea, and cerium nitrate hexahydrate were used. The optimum reaction time and temperature were 39 h and 120 °C, respectively. Surface area values between 30 and 166 m^2^/g were reported. Large-scale CeO_2_ nanowires were prepared using the sol-gel method process at 600 °C by Wu et al. where porous anodic alumina template and cerium nitrate hexahydrate were used [27]. Micro- and nano-sized spheres have been obtained with different precursor materials. For example, Liu et al. fabricated mono-dispersed CeO_2_ hallow nanospheres at 300 °C; with an average size in the range of 50–200 nm, surface area value of 61 m^2^/g and pore size of 50 nm were obtained [28]. In this synthesis, tetrahydrate sulfate cerium and ammonium were employed. In addition, CeO_2_ microspheres were obtained at 600 °C by Feng et al.; cerium nitrate hexahydrate, potassium sulfate, and nitric acid were used for this purpose; surface area value of 15.6 m^2^/g was reported [29].

In a previous work, hierarchical 3D architectures of CeO_2_ were obtained at 400 °C by the microwave-assisted coprecipitation method. For this synthesis process, these were used ceric ammonium nitrate and formic acid as precursor materials. The morphology of the samples obtained from 170 to 600 °C was composed of hollow microspheres, with a size between 1.5 and 10 μm. These powders were used to fabricate CO gas sensor devices. As a general trend in gas sensing characterization, no steady state was observed and the response time was at least 20 s [30].

In the present work, to study the effect on microstructure and synthesis temperature, a similar synthesis method was employed to prepare CeO_2_, but cerium nitrate was used instead of ceric ammonium nitrate. The first aim in this work was to get CeO_2_ at lower temperature and with this an increase in the surface area, which will be seen in the sensor device performance. The sensing characterization was carried out at lower temperature than in the previous work. The effect of the cerium nitrate in the morphology was analyzed by FESEM. Moreover, the surface area and pore size were determined by the BET method. The gas sensing behavior was analyzed using an alternating current (AC), with different operational temperatures, frequencies, and gas concentrations. The second aim was to get stable electrical measurements; both aims were achieved.

## Methods

All reagents used in this work were analytical grade and were used without further purification. The process to synthesize nanostructured CeO_2_ microspheres was the following: 0.76 g of cerium nitrate was dissolved into 10 ml of concentrated formic acid (>95%), under strong stirring. The resultant suspension was stirred for 24 h, at room temperature. Later, the solvent was evaporated intermittently using a domestic microwave oven, in steps of 40 s. The resulting material was dried overnight at 100 °C and later calcined to 200 and 300 °C for 5 h, in air.

The crystal structure was analyzed by X-ray powder diffraction (XRD), using an Empyrean (PANalytical) diffractometer, with Cu-K_α_ radiation (*λ* = 0.15418 nm), and diffuse reflectance FT-IR spectroscopy, using a Perkin-Elmer GX3 spectrometer, with samples dissolved in KBr. The surface area was determined by the Brunauer–Emmett–Teller (BET) method, and the pore size distribution was calculated by the Barrett–Joyner–Halenda (BJH) method, using desorption branch. Before measurements, the sample was vacuum degassed at 200 °C, for 12 h, using a TriStar II Plus apparatus.

The morphology of the samples was investigated by FESEM (Hitachi, SU8010) and HRTEM (FEI, Tecnai F30) with an accelerating voltage of 200 kV. No special treatment was applied for the FESEM analysis. The sample for TEM characterization was prepared by placing a drop of colloidal solution on carbon-coated copper grid and dried at room temperature.

**Fig. 1:**
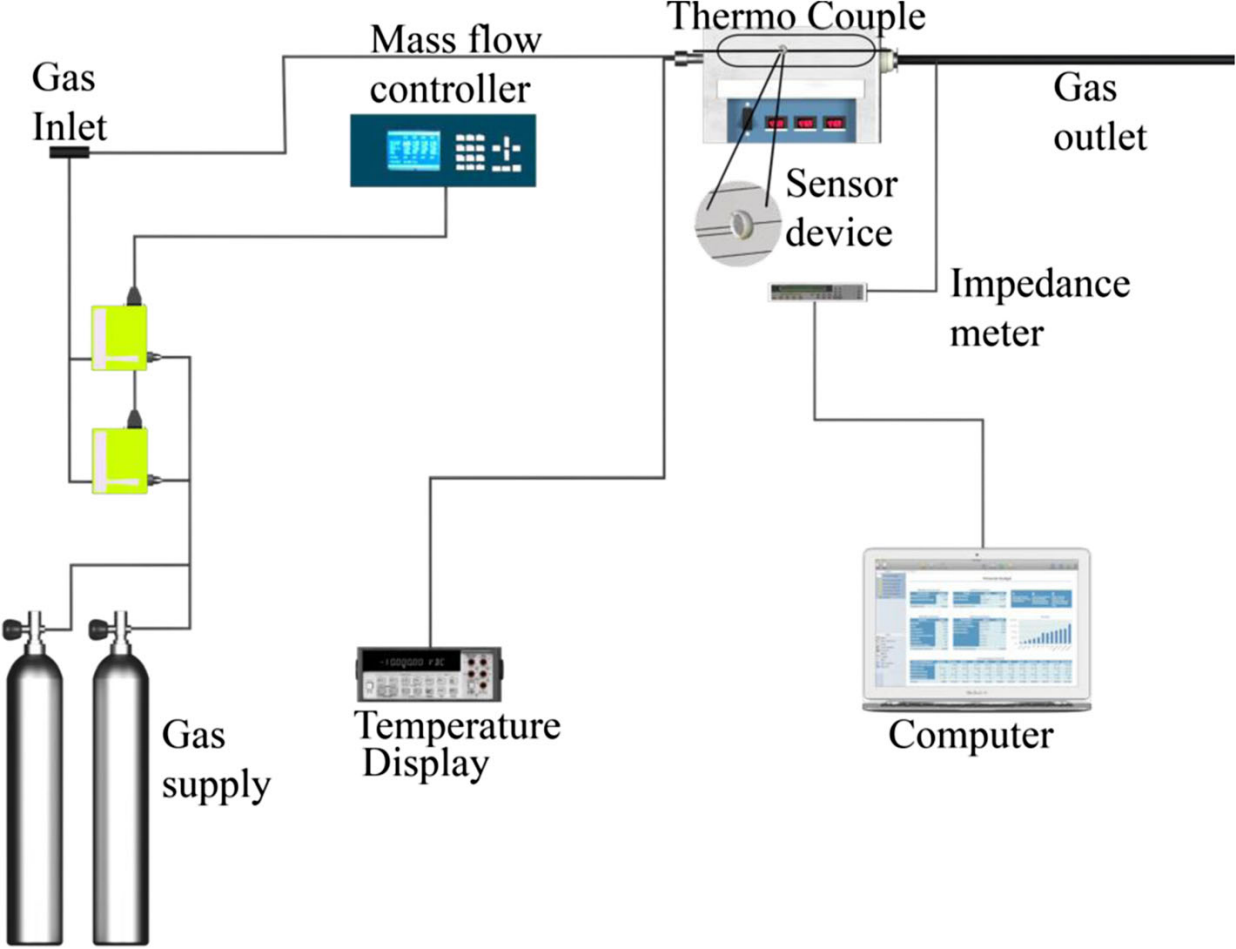
Scheme for gas sensing characterization

The gas sensing characterization was performed on thick films made with the CeO_2_ microspheres. The sensor device was placed into a tube-type furnace, which served as a test chamber. Impedance measurements were performed using an LCR meter (Agilent 4263B). The test gases were CO (99.3%) and air (base gas), both extra dry, purchased from Infra, México. A mass flow control system (MKS Instruments, 647C) was used to introduce the gases into the test chamber. The response (*t*_res_) and recovery (*t*_rec_) times are defined as the times elapsed to reach 90% of the change in electrical impedance, upon exposure to the test and the base gas, respectively. Figure 1 displays a scheme of the experimental setup.

## Results and Discussion

**Fig. 2:**
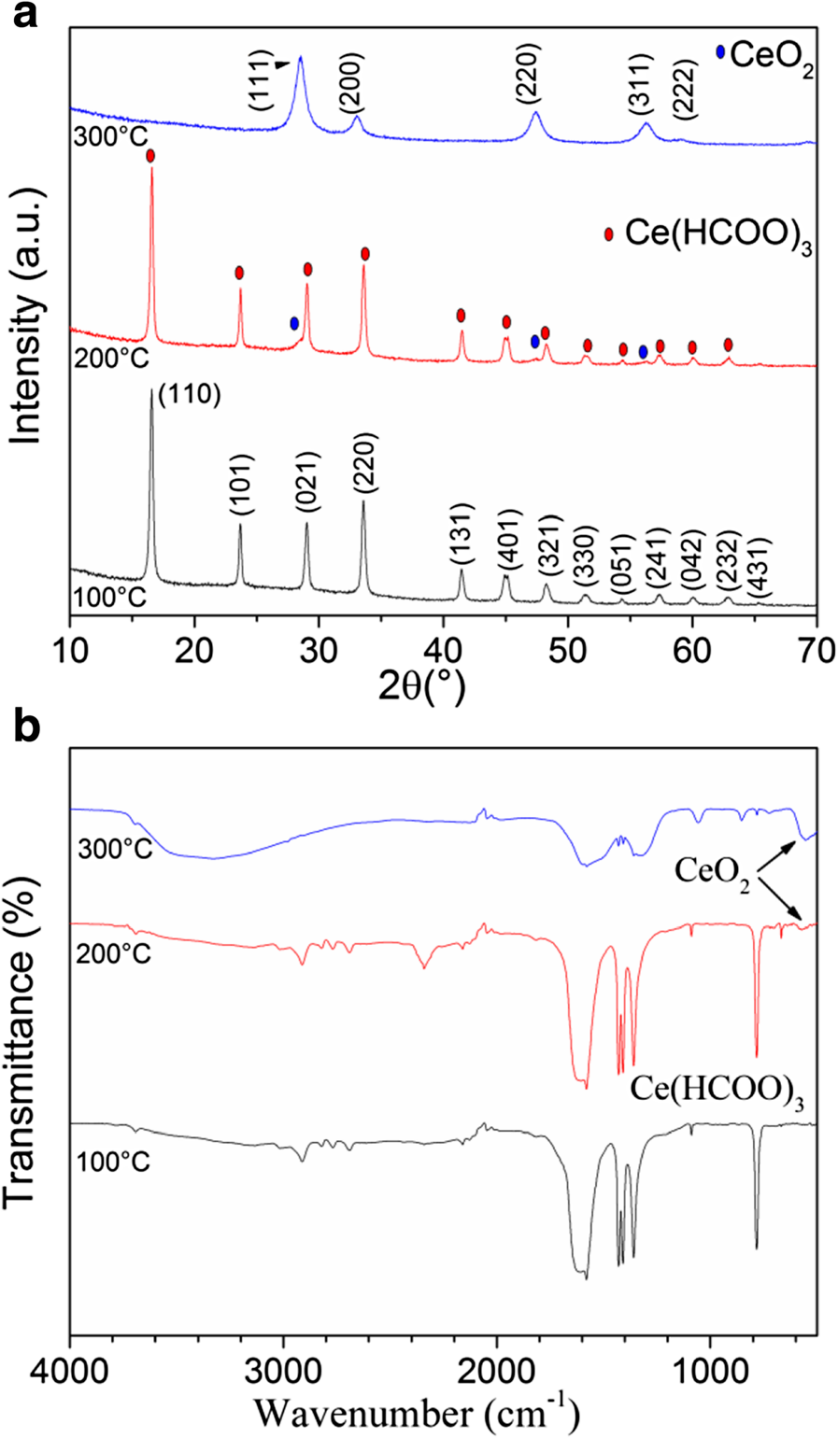
**a** XRD patterns and **b** FT-IR spectra for the precursor powder calcined at different temperatures

Figure 2a shows XRD patterns of samples calcined from 100 to 300 °C. At 100 °C, single-phase cerium formate (JCPDF No. 49.1245) was formed. This compound crystallizes in a trigonal crystal structure, with cell parameters *a* = *b* = 10.682 Å and *c* = 4.109 Å. Calcination at 200 °C produced mainly cerium formate, but diffraction lines of CeO_2_ (JCPDF No. 34-0394) can be noticed at 2(*θ*) = 28.54°, 47.48°, and 56.33°. At 300 °C, diffraction peaks are located at 2(*θ*) = 28.53°, 33.03°, 47.42°, 56.29°, and 59.02° which are related to the planes (111), (200), (220), (311), and (222), which correspond to CeO_2_, with cubic fluorite-type structure and space group Fm-3m. The lattice parameter previously reported for cubic CeO_2_ is *a* = 5.411 Å, whereas in this work, a calculated value of 5.41417 Å was obtained. According to Ferreira et al., the lattice expansion of CeO_2_ can be attributed to the reduction of Ce^4+^ to Ce^3+^, which involves a decrease in electrostatic force [31].

FT-IR spectra of calcined samples are shown in Fig. 2b. Samples calcined at 100 and 200 °C show broad absorption bands of the asymmetric and symmetric stretching vibrations of OHO, placed at 3080–3700 cm^−1^. The IR absorption peaks at 2900 cm^−1^ are assigned to C–H stretching vibrations. The bands at 1650–1540 cm^−1^ and 1447–1340 cm^−1^ correspond to the asymmetric and symmetric stretching vibrations of the bound carboxylic groups (COO^−^). And the band at 780 cm^−1^ is related to the stretching vibrations of C–C groups. The spectra obtained from samples annealed at 200 and 300 °C show the characteristic band of Ce–O stretching vibrations, located at around 500 cm^−1^ [32]. The spectrum of a sample calcined at 300 °C shows the presence of a small amount of organic matter.

**Fig. 3:**
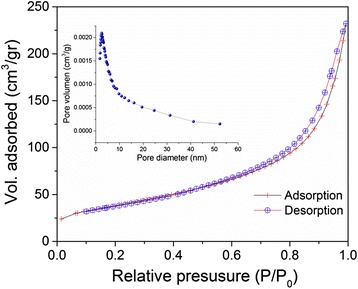
Nitrogen adsorption–desorption isotherms and Barret–Joyner–Halenda (BJH) pore size distribution of nanostructured CeO_2_ microspheres

Figure 3 shows adsorption–desorption isotherms and BJH pore size distribution for nanostructures CeO_2_ microspheres. The isotherm can be ascribed as type IV, which means that is associated to capillary condensations according to IUPAC classification with type H3 hysteresis which is characteristic of the mesoporous material with slit-shaped pores [33]. The adsorption increased from low pressure of about 0.01 to 0.8, and it was followed by a sharp rise from 0.8 and above due to substantial interparticle porosity [34]. The BET surface area was calculated at 137 m^2^/g, whereas the average pore diameter was 9 nm. Similar results (155 m^2^/g, 3.5 nm) were reported by Hung et al. where a tri-block copolymer was used as template [35].

**Fig. 4:**
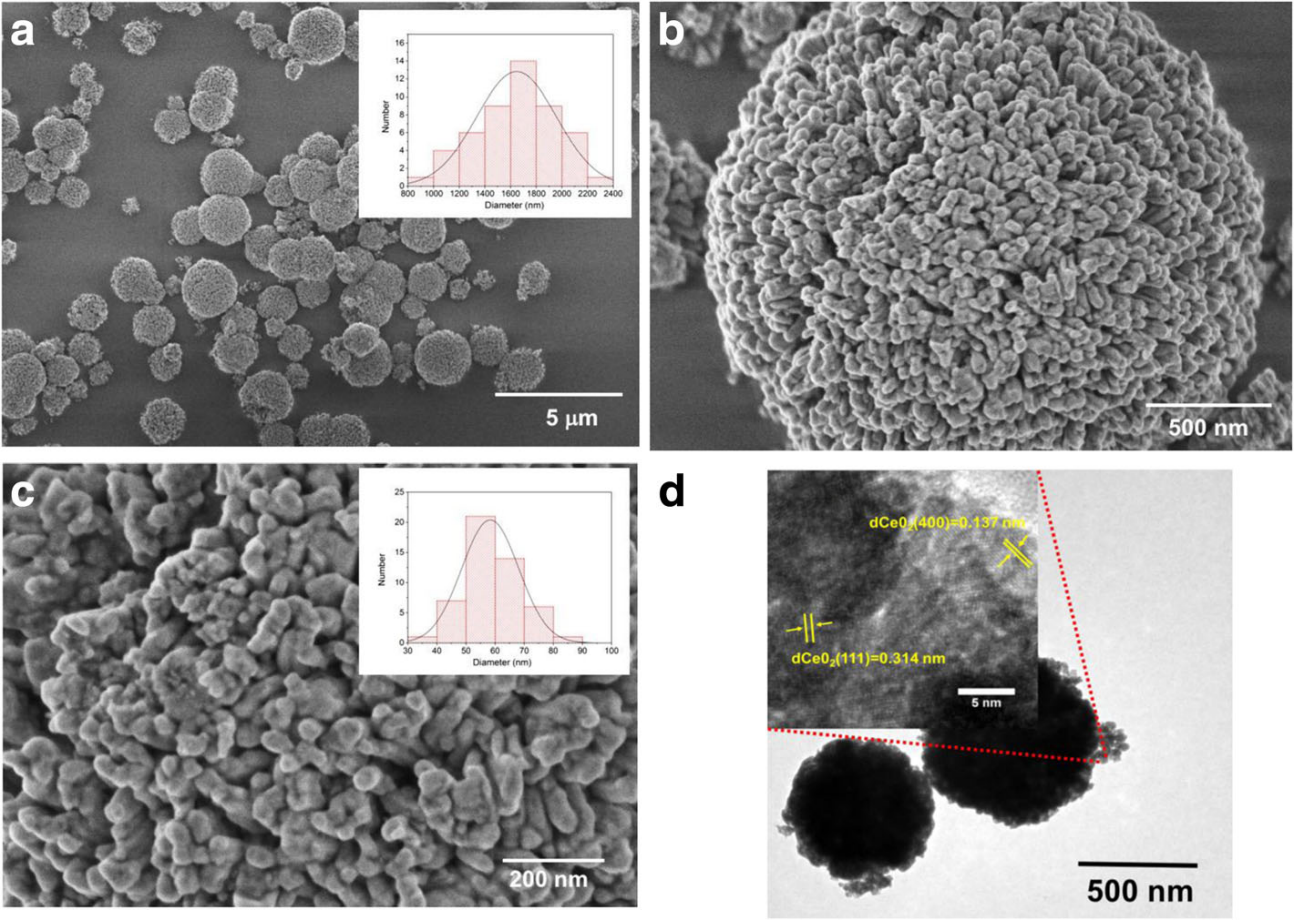
**a**–**c** FESEM at different magnificatios. **a** and **c** shows microspheres and grain size distributions histogram. **d** HRTEM images of CeO_2_ microspheres calcined at 300 °C

Figure 4a–c shows FESEM images of a CeO_2_ sample calcined at 300 °C. Figure 4a displays porous microspheres with diameters between 0.8 and 2.4 μm (inset of figure). Figure 4b, c also shows detailed characteristics of the morphology of CeO_2_ microspheres. Extensive porosity among nanoparticles with size between 30 and 90 nm can be noticed (inset of figure). HRTEM image (Fig. 4d) shows individual CeO_2_ microspheres, with nanoparticles on their surface. The inset of this image displays numerous lattice planes, which confirms the crystallinity of the sample. Besides, the interplanar distance of planes (400) and (111) was determined, resulting in 0.13 and 0.31 nm, respectively.

The CO sensing characterization was carried out by measuring the transient response where electrical impedance was used. The electrical impedance (*Z*) can be written in the polar or Cartesian forms by means of the Eq. (1):

1 $$ Z=\left| Z\right|{e}^{i\varphi}= R+ i X $$

where |*Z*| is the magnitude of impedance, *i* is defined as the imaginary unit, *φ* = arg(*Z*), *R* is the resistance, and *X* is the reactance. An advantage of recording |*Z*| vs. time graphs, over direct current measurements, is that the gas response could be analyzed using different frequencies. Response is defined by Eq. (2):

2 $$ R=\frac{\varDelta \left| Z\right|}{\left|{Z}_{\mathrm{air}}\right|}=\frac{\left|{Z}_{\mathrm{air}}\right|-\left|{Z}_{\mathrm{CO}}\right|}{\left|{Z}_{\mathrm{air}}\right|} $$

**Fig. 5:**
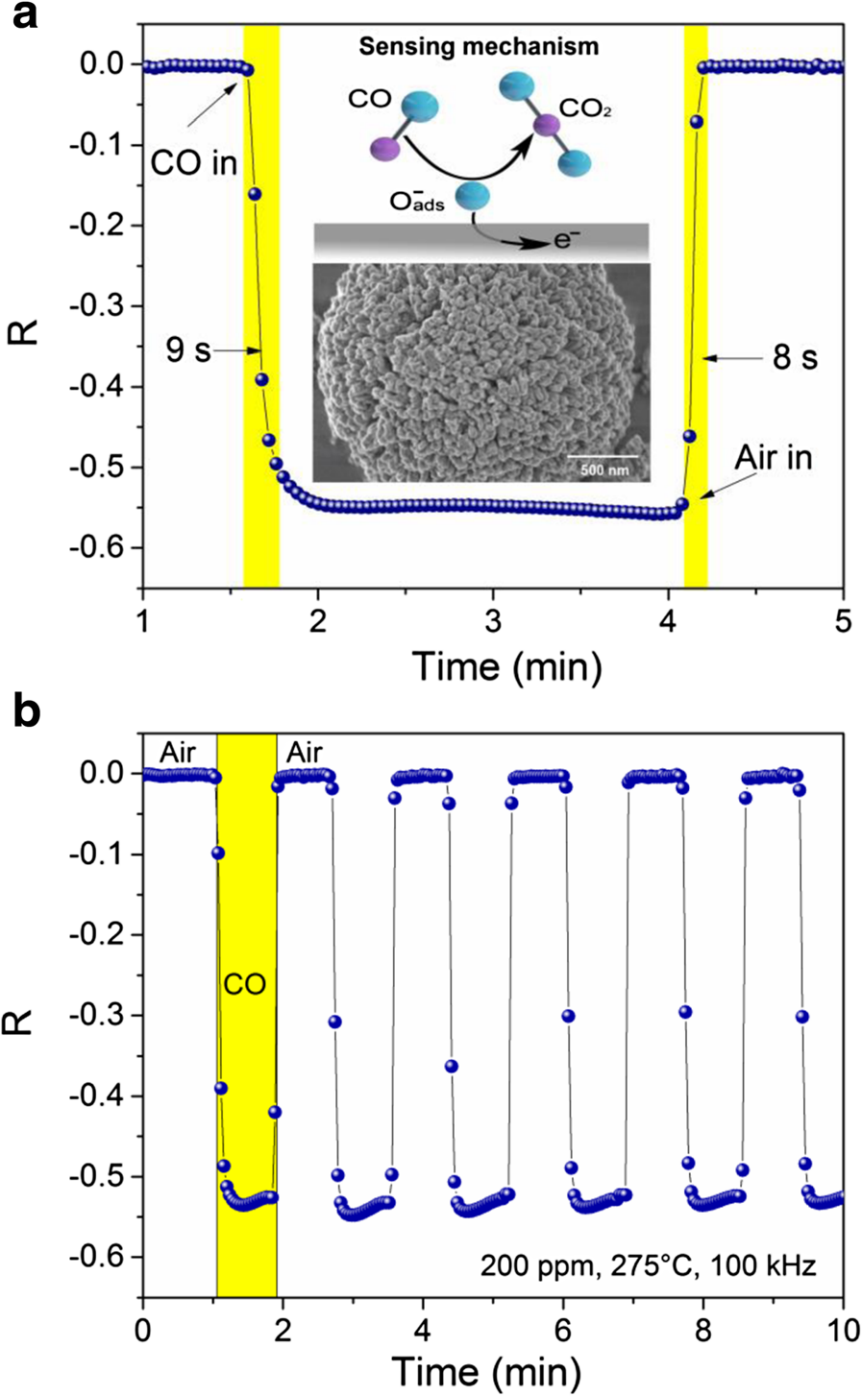
Carbon monoxide gas sensing response vs. time with a concentration of 200 pm, operate at 275 °C and 100 KHz. **a** Single cycle to determine response and recovery time. **b** Several cycles to determine repeatability in the detection of CO (shorter cycles)

Figure 5a, b shows the transient response obtained in air and 200 ppm CO, using an applied frequency of 100 kHz, at 275 °C. As it is also possible to note a decrease in the magnitude of the impedance (|Δ*Z*|) occurred once CO (reducing gas) was injected, and upon the introduction of air, |Δ*Z*| returned to its original value, which corresponds to an n-type semiconductor material. The average variation of |Δ*Z*| was 0.3 MΩ, where the steady state can be observed. The steady state occurs when the process of charge transfer between gas and sensor device and continues until equilibrium is reached [36, 37]. The gas sensing response is based on surface reaction between adsorbed oxygen and the gas to be detected. According to what was described by Gong et al., the interaction of gas to be sensed and the thick film includes two steps [38]. In the first step, oxygen from the ambient is adsorbed on the surface of the film and extracts electrons from the material. This depends on the operating temperature as follows:

3 $$ {\mathrm{O}}_{2(ads)}+{e}^{-}\leftrightarrow {0}_{2(ads)}^{-}, T<100\ {}^{\circ}\mathrm{C} $$

4 $$ {\mathrm{O}}_{2(ads)}^{-}+{e}^{-}\leftrightarrow 2{O}_{(ads)}^{-}, T=100-300\ {}^{\circ}\mathrm{C} $$

5 $$ {\mathrm{O}}_{(ads)}^{-}+{e}^{-}\leftrightarrow {\mathrm{O}}_{2(ads)}^{-},\  T>300\ {}^{\circ}\mathrm{C} $$

In the second step, CO molecules can react with oxygen species on surface of the sensor device which reduce the electrical resistance. It can be described as:

6 $$ 2\mathrm{C}\mathrm{O}+{\mathrm{O}}_2^{-}\to 2\mathrm{C}{\mathrm{O}}_2+{e}^{-},\  T<100\ {}^{\circ}\mathrm{C} $$

7 $$ \mathrm{C}\mathrm{O}+{\mathrm{O}}^{-}\to \mathrm{C}{\mathrm{O}}_2+{e}^{-}, T=100-300\ {}^{\circ}\mathrm{C} $$

8 $$ \mathrm{C}\mathrm{O}+{\mathrm{O}}_2^{-}\to \mathrm{C}{\mathrm{O}}_2+2{e}^{-},\  T>100\ {}^{\circ}\mathrm{C} $$

From Fig. 5a, a response time (*t*_res_) of 9 s was determined, which corresponds to the elapsed time measured at the 90% of the variation of Δ|*Z*| in the tested gas. When CO is removed, Δ|*Z*| returned to its original value; recovery time (*t*_rec_) of 8 s was calculated analogous to *t*_res_. The recovery process is determined by two steps, oxygen re-adsorption from the ambient at the surface and re-oxidation of the oxide [39]. Nanometric grain size and large grain boundary could enhance the rapid recovery time and the Δ|*Z*| value due to the adsorption of the oxygen on the surface. Figure 5b displays repeatability in the detection of CO, and this can be observed with the Δ|*Z*| value after several cycles, which indicates that |Δ*Z*| returns to its initial value and the CO adsorbed on the surface was reversible. A previous work [30] has shown that the response time was 47 s, at 250 °C, 100 Hz and 300 ppm of CO, and the results did not show a steady-state. For a comparative purpose, Izu et al. [40] prepared two samples of CeO_2_. The first named core–shell nanoparticles (with Au 4 wt%), and the second named precipitated nanoparticles. The average particle size was 30 nm for the sensor devices. The gas sensing performance was carried out at 450 °C. The results show that the sensor prepared using the core–shell nanoparticles exhibits a response time of 46 s whereas the response time of the sensor fabricated using the precipitated nanoparticles was 49 s as an average, when CO concentrations varies from 1% to 5000 ppm. The recovery time reported for the measurements was an average of 50 s. Durrani et al. [41] prepared thin film of tin oxide mixed with cerium oxide by physical vapor depositions; the films were annealed at 500 °C. When the film was exposed to 500 ppm of CO at 430 °C, the response time was 26 s and the recovery time was 30 s. The morphology reported was a well-separated conical nano columnar structure. In both works, the response and recovery time are much larger that obtained in this work where nanostructured CeO_2_ microspheres was used to prepared sensor device.

**Fig. 6:**
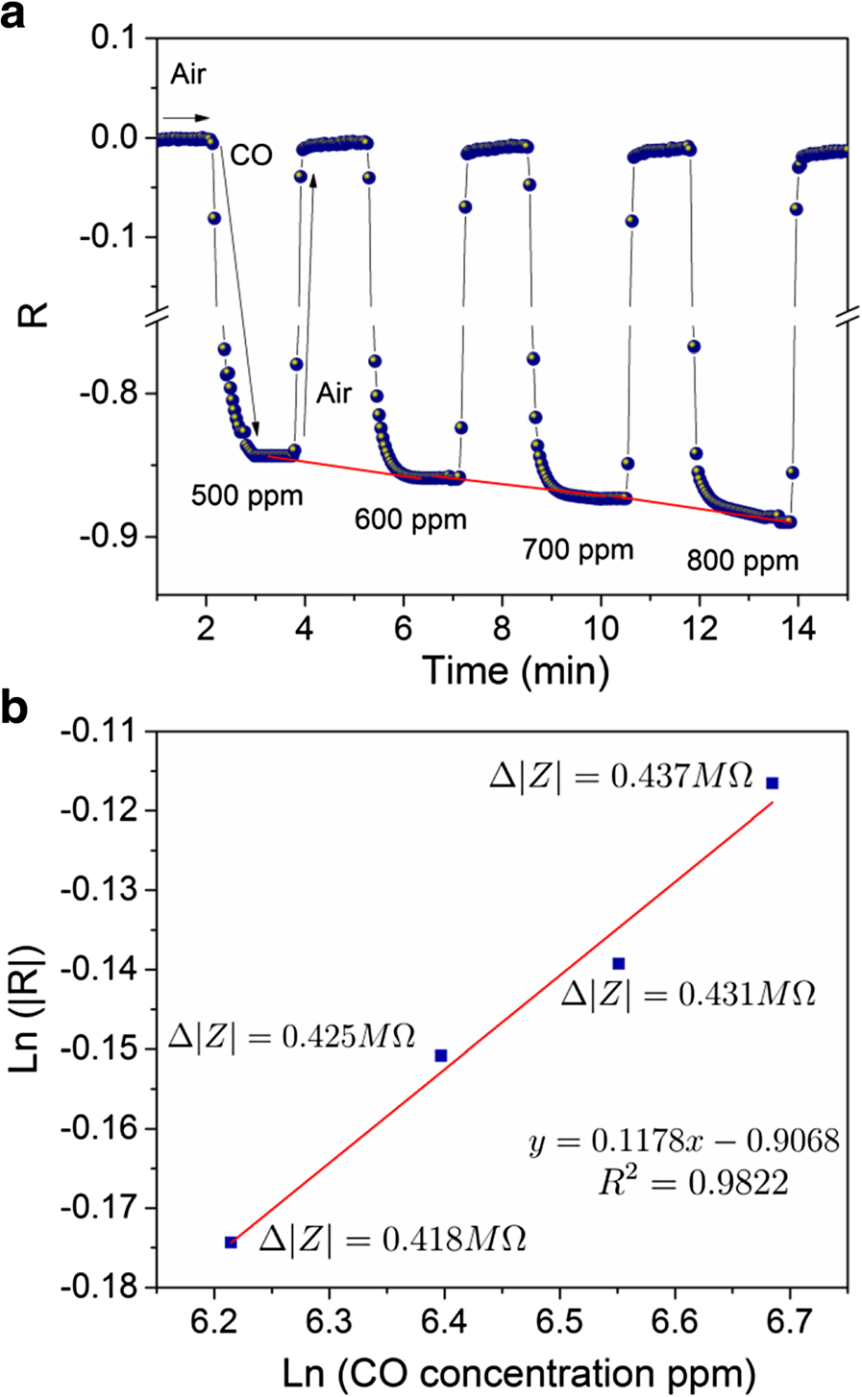
**a** The effect of CO gas concentrations from 500 to 800 ppm in sensor response operated at 275 °C and 100 kHz. **b** Adsortion isotherm plot from data of Fig. 6a where |Z| is the magnitud of impedance

The sensitivity as a function of CO gas concentrations from 500 to 800 ppm for the CeO_2_ thick film at 275 °C and 100 kHz is shown in Fig. 6a. From this graph, a non-linear variation of Δ|*Z*| with gas concentration can be noticed (bottom line). This behavior can be described by the Freundlich adsorption isotherm: *N = kC* ^*γ*^; where *k* and *γ* are constants and *N* and *C* are the concentrations of absorbed oxygen and CO, respectively [42]. Since *γ* is smaller than 1, the adsorption of oxygen depends weakly on *C.* As is possible to notice, when the CO gas is removed from the chamber test, the Δ|*Z*| value recovers its initial value in each cycle, which means that the CeO_2_ sensor device has a good response for different CO concentrations. Figure 6b shows an adsorption isotherm plot from data of Fig. 6a.

**Fig. 7:**
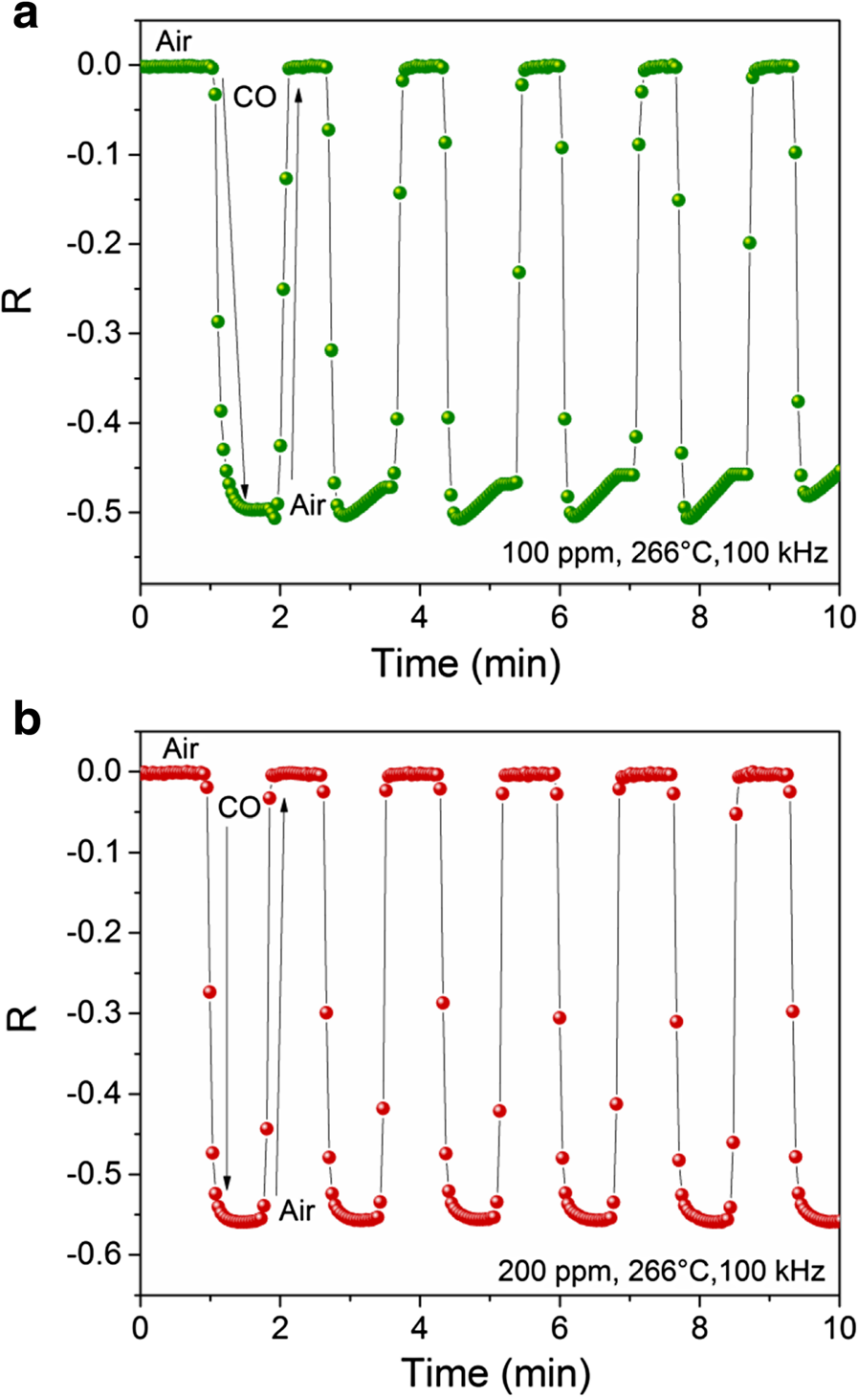
Sensor response to 100 (**a**) and 200 (**b**) ppm of CO gas operated at 266 °C and 100 kHz

To determine the response of the sensor device, several measurements were carried out at different temperatures, gas concentrations, and frequencies. Figure 7a, b compares the CO sensing response measured at 266 °C and 100 kHz, with 100 and 200 ppm, respectively. When 100 ppm was supplied, the average value of Δ|*Z*| was 0.25 MΩ and *t*_res_ = 10 s, whereas with 200 ppm, the values were Δ|*Z*| = 0.28 MΩ and *t*_res_ = 11 s. The pore size distribution in this material corresponds to mesoporous; then, the gas diffusion through a porous material depends on the pore size, and this can be described by Knudsen diffusion [43]:

9 $$ {D}_k=\frac{4 r}{3}\sqrt{\frac{2 RT}{\pi M}} $$

where *T* is the temperature, *r* pore radius, *M* molecular weight of the diffusing gas, and *R* gas ideal constant. When molecules of the target gas are in contact with the porous surface, it can react. The reaction is assumed to follow a first-order kinetic function. If Knudsen diffusion and the last one are assumed, a well-known diffusion equation can be formulated:

10 $$ \frac{\delta {C}_A}{\delta t}={D}_k\frac{\delta^2{C}_A}{\delta {x}^2}- k{C}_A $$

where *C*_*A*_ is the concentration of the target gas, *t* is time, *D*_*k*_ is Knudsen diffusion coefficient, *x* is distance (depth) from the top surface of the sensing layer, and *k* is rate constant. This means that the concentration of the oxygen in the film should decrease due to the surface reaction. *D*_*k*_ can be expressed as a function of *C*_*A*_. Liu et al. [44] in a theoretical approach tested different gas concentrations and found an expression to response time (*t*_res_ = 0.85 *L* ^2^/*D*). In these works, it was concluded that response time is dominated by sensor thickness (*L*) and diffusion coefficients (*D*); the last one depends on the gas concentration. The response times for 100 and 200 ppm are very closed and could follow this relation. However, as the difference is small, it could be attributed to experimental error. However, a different behavior was noticed. From Fig. 7a, an absence of the steady state was observed; the opposite occurs for Fig. 7b. For the two gas concentrations tested (100 and 200 ppm), when CO gas was pumped out, Δ|*Z*| recovered its initial value. This behavior was described by Wolkenstein in [45]. A general description could be as follows: at the beginning of the process, there are not enough molecules of CO available to react with the surface oxygen adsorption sites. This changes after some seconds, when the CO molecules get adsorbed. Figure 7a shows in a graphical manner the described behavior. After that initial stage, the steady state is observed. In contrast, when CO concentration increases, more molecules react with oxygen vacancies. This is illustrated in Fig. 7b. This allows to observe the steady state when 200 ppm of CO was supplied and then a higher response is obtained. In both cases, the desorption is completely irreversible, which indicates that *R* returns to the original value. With this, neutral and charged particles are removed from the surface during desorption. Even more, this value was constant after several cycles which indicate a long-term stability.

**Fig. 8:**
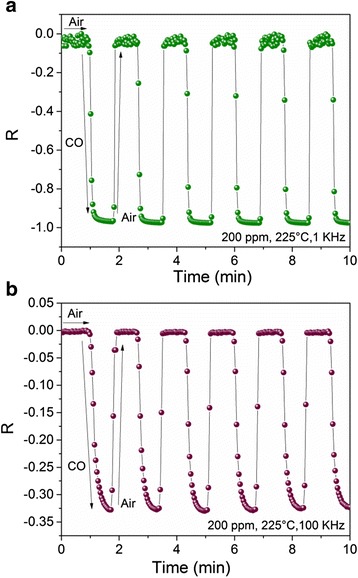
Variations of *R* with time measured in 200 ppm (225 °C) at **a** 1 and **b** 100 kHz

To study the frequency effect over the sensor device response, the samples were tested at 1 and 100 kHz; the results are shown in Fig. 8a, b, respectively. For these measurements, an operating temperature of 225 °C, a concentration of 200 ppm of CO gas, and a time of 10 min of exposition to this gas were used. From Fig. 8a, it is observed that after several cycles, the average value of Δ|*Z*| = 43 MΩ and the *t*_res_ = 12 s which reveals the steady state. When 100 kHz is applied, Δ|*Z*| was estimated in 0.17 MΩ with a *t*_res_ = 13 s. As it is possible to notice from Fig. 8, the average response of the sensor decreases about two orders of magnitude when 100 kHz was applied. This can be understood as a low-pass filter [46], and the frequency response function can be expressed as:

11 $$ R\left( i\omega \right)=\frac{R_0\left( i\omega \right)}{1+\tau \left( i\omega \right)} $$

where *R*_0_ is the initial response, *ω* is the frequency, *τ* is the system time constant, and *i* is the imaginary unit. In addition, a decrease of noise was observed and this noise is known as flicker noise or 1/f noise. In addition, the effect of the temperature operation over response time was noticed. When measurements were carried out with the same conditions (100 kHz, 200 ppm; Figs. 7b and 8b), the response time increased slightly when the operating temperature decreased (266 to 225 °C, respectively). This effect was widely studied by Helwig et. al and is related with the changes in activation energy on the surface during adsorption and desorption process [47].

**Fig. 9:**
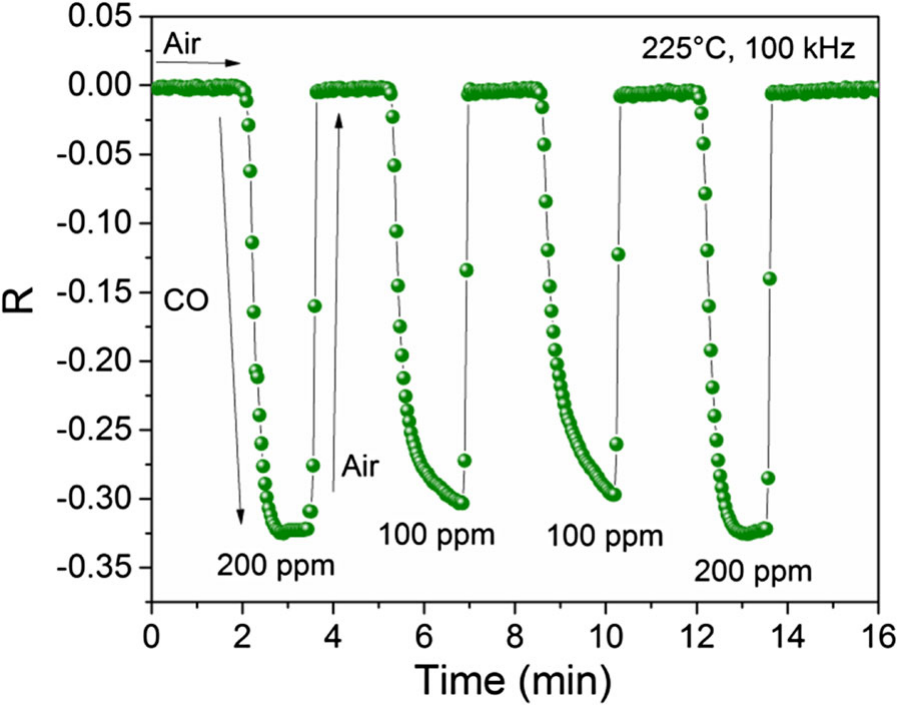
Reproducibility of the gas sensing response when 100 and 200 ppm of CO were supplied at 100 kHz and 225 °C

The reproducibility of gas sensing response at 100 kHz and 225 °C when different gas concentrations were supplied is shown in Fig. 9. In the first cycle, 200 ppm of CO was applied, followed by two 100-ppm cycles, and finally, a 200-ppm cycle was tested. After these cycles, Δ|*Z*| remains constant, and this value for 200 ppm was 0.17 MΩ whereas for 100 ppm was estimated in 0.15 MΩ. When the CO was removed from the chamber test, Δ|*Z*| recovers its initial value, which indicates a good response and reproducibility for different gas concentrations.

## Conclusions

Nanostructured CeO_2_ microspheres with large surface area and mesoporosity were synthesized by the coprecipitation method at low calcination temperature. Compared to previous results obtained from CeO_2_ where hierarchical architectures were obtained, in our results, a narrower particle size distribution was obtained. Also, in this work, microspheres also displayed a uniform nanostructured surface morphology. The gas sensor device based on as-prepared CeO_2_ microspheres exhibited more stable electrical measurements, which were observed by the development of the steady state; slightly upper response time (*t*_res_) was obtained using a lower operating temperature and a good recovery performance. The improvement of the CO sensing response can be attributed to a larger porosity and less particle agglomeration of CeO_2_ microspheres.
